# Robot-Assisted Arm Training in Stroke Individuals With Unilateral Spatial Neglect: A Pilot Study

**DOI:** 10.3389/fneur.2021.691444

**Published:** 2021-07-08

**Authors:** Ze-Jian Chen, Ming-Hui Gu, Chang He, Cai-Hua Xiong, Jiang Xu, Xiao-Lin Huang

**Affiliations:** ^1^Department of Rehabilitation Medicine, Tongji Hospital, Tongji Medical College, Huazhong University of Science and Technology, Wuhan, China; ^2^World Health Organization Cooperative Training and Research Center in Rehabilitation, Wuhan, China; ^3^State Key Lab of Digital Manufacturing Equipment and Technology, Institute of Rehabilitation and Medical Robotics, Huazhong University of Science and Technology, Wuhan, China

**Keywords:** stroke, rehabilitation, robotics, unilateral spatial neglect, upper extremity

## Abstract

**Background:** Robot-assisted arm training (RAT) is an innovative exercise-based therapy that provides highly intensive, adaptive, and task-specific training, yet its effects for stroke individuals with unilateral spatial neglect remain to be explored. The study was aimed to investigate the effects of RAT on unilateral spatial neglect, arm motor function, activities of daily living, and social participation after stroke.

**Methods:** In a pilot randomized controlled trial, individuals with unilateral spatial neglect after right hemisphere stroke were equally allocated to intervention group and control group, 45-min training daily, 5 days/week, for 4 weeks. Outcome measures included the Behavioral Inattention Test-conventional section (BIT-C), Catherine Bergego Scale (CBS), Fugl-Meyer Assessment for Upper Extremity (FMA-UE), Modified Barthel Index (MBI), and World Health Organization Disability Assessment Schedule Version 2.0 (WHODAS 2.0).

**Results:** From November 2018 to February 2021, 20 stroke patients (mean age 47.40 ± 8.47) were enrolled in the study. Robot-assisted arm training was feasible and safe for individuals with unilateral spatial neglect. Both groups had significant improvements in all outcome measures. Participants assigned to RAT therapy had significantly greater improvements in BIT-C (difference, 7.70; 95% CI, 0.55–14.85, *P* = 0.04), FMA-UE (difference, 5.10; 95% CI, 1.52–8.68, *P* = 0.01), and WHODAS 2.0 (difference, −7.30; 95% CI, −12.50 to −2.10, *P* = 0.01). However, the change scores on CBS and MBI demonstrated no significance between the groups.

**Conclusion:** Our findings provide preliminary support for introducing robot-assisted arm training to remediate unilateral spatial neglect after stroke. The training program focusing on neglect of contralateral space and affected upper extremity may be effective in neglect symptoms, motor function recovery, and social participation, while not generalizing into improvements in activities of daily living.

**Clinical Trial Registration**: Chinese Clinical Trial Registry (http://www.chictr.org.cn/) on 17 October 2019, identifier: ChiCTR1900026656.

## Introduction

Unilateral spatial neglect (USN) is the common cognitive impairment following stroke and refers to a failure to report, respond, or orient to stimuli presented to the side opposite the brain lesion. USN is present in at least 30% of stroke survivors and nearly half of individuals with right hemisphere stroke (1, 2). Neglect symptoms may persist into the chronic stroke stages, significantly interfere the participation in rehabilitation programs, and lead to poorer outcomes after stroke (3, 4). Lack of sufficient evidence for effectiveness of pharmacological medication according to a Cochrane systematic review, the state of the art USN management mainly relies on rehabilitation interventions (5, 6).

Various rehabilitation interventions have been developed for USN and generally divide into two categories: top-down (remedial) and bottom-up (compensatory) approaches (6). Top-down approaches, aimed at teaching patients strategies to compensate for their neglect deficits voluntarily, require full cooperation from the patient to be aware of the neglected space, such as visual scanning and mental imagery (7, 8). Bottom-up approaches, designed to adapt the external environment or apply sensory stimulation, focus on remediating patients' perception of neglected space, such as prism adaptation (9), limb activation therapy (10), optokinetic stimulation (11, 12), feedback training (13), neck muscle vibration (14), trunk rotation (15), and eye patching (16). However, there is only limited evidence and effects of these methods on neglect symptoms and functional performance with current methods (17, 18). Meanwhile, non-invasive brain stimulation (19) or combination of multiple methods in a patient-oriented protocol has shown promise in strengthening the clinical outcomes (20–22).

Robot-assisted arm training (RAT) is an innovative exercise-based therapy that provides highly intensive, adaptive, and task-specific upper limb training with sensorimotor stimulation for stroke individuals (23, 24). In addition, audiovisual cues and feedback information about performance are available for patients to increase motivation in a virtual environment provided by human–robot interaction (25). Extensive research has denoted that RAT could improve arm muscle strength, upper extremity function, and activities of daily living (ADL) after stroke (23, 26). However, the general applicability of these results to stroke patients with USN remains indeterminate.

In a case series study, Varalta et al. reported that robot-assisted hand training showed potentials in the recovery of USN after stroke (27). Training with a hand rehabilitation glove that provides computer-controlled, repetitive, passive mobilization of the fingers, stroke patients had improvement in visuospatial exploration, attention, and fingertip dexterity. However, due to the limited sample size and the conflicting results with Choi et al. it is uncertain whether RAT could be implemented in stroke patients with USN (28). Besides, the devices in previous studies were wrist-hand or planar robot, thus supporting motions with a limited degree of freedom in a low dimensional space. Compared with end-effector, exoskeleton robots can apply quantified torque to multiple joints, allowing considerable ranges of motions in three-dimension space (29).

To our best knowledge, the effects of exoskeleton-driven RAT, according to the International Classification of Functioning, Disability and Health (ICF) framework (30), for stroke individuals with USN remain to be investigated. Therefore, the aim of this pilot study was to evaluate the preliminary effects of RAT on USN, arm motor function, ADL and social participation after stroke.

## Methods

### Study Design

This study was an assessor-blinded, prospective, pilot randomized controlled trial (RCT) with two parallel arms: RAT group and conventional therapy (CT) group. The Clinical Trials Ethics Committee of Huazhong University of Science and Technology has given ethical approval on October 24, 2018. Trial registration is on Chinese Clinical Trial Registry from the WHO registry network (ChiCTR1900026656).

### Participants

We screened and enrolled eligible patients from November 2018 until February 2021. The patients received cerebral reperfusion therapy for ischemic stroke or hematoma evacuation for hemorrhagic stroke initially in the stroke unit, and were referred to the Department of Rehabilitation Medicine. Prior to study participation, written informed consents were obtained from all participants according to the latest Declaration of Helsinki. Inclusion Criteria included: (a) age 18–80; (b) clinical diagnosis of right hemisphere stroke (stroke onset from 2 weeks to 6 months); (c) Fugl-Meyer assessment of the upper extremity (FMA-UE) score 8–47; and (d) presence of USN defined by scoring of any item lesser than its cutoff value of the Behavioral Inattention Test conventional section (BIT-C). Exclusion criteria included: (a) not first-ever stroke; (b) other current significant impairments, for example, visual impairment, fixed contracture, shoulder subluxation; (c) diagnosis likely to interfere with rehabilitation or outcome assessments, for example, traumatic brain injury, epilepsy; and (d) unable to understand the intervention because of aphasia or other cognitive impairments (9).

### Randomization and Blinding

Participants who complied with the eligibility criteria and signed the written informed consent were evaluated by the researchers for enrollment. At enrollment, information was collected including demographic data, stroke information, Montreal Cognitive Assessment, hand dominance, comorbidity, and functional measurements. As measured by the Edinburgh Handedness Inventory, all participants in this pilot study were right-handed (31). A researcher who was not involved in the recruitment, evaluation, or treatment performed the randomization procedure through a computer-generated random number table. Because the training approaches differed greatly between groups, it was not feasible to blind the subjects, therapists, or physicians participating in the study. Therefore, an independent evaluator blinded to the randomization procedure and group allocation completed all the outcome measures at baseline and after intervention.

### Interventions

Participants in the RAT group received RAT (Armule®, Intelbot intelligent machine Co., Ltd, Wuhan, China) (32) for remediating patients' neglect of contralateral space and affected upper extremity supervised by a therapist. The exoskeleton allowed complex movements of the upper extremity in a three-dimensional workspace with 7 degrees of freedom involving shoulder (flexion/extension, abduction/adduction and internal/external), elbow (flexion/extension, forearm supination/pronation), and wrist (flexion/extension and ulnar deviation/radial deviation). When receiving robotic therapy, patients sat in a height-adjustable chair in front of the exoskeleton and looked at the computer monitor connected to the robotic device. Linkages between patients and the Armule were custom-fitted based on arm length and circumference. In addition, motion sensors were placed in the linkage cuffs of upper arm and forearm to detect the patient's movement intention. The robotic programs in this study were adapted to apply training for motor impairment and USN simultaneously by increasing left-side Armule sensorimotor interaction with the patients. Each training session consisted of 15-min passive mode and 30-min assist-as-need mode. During passive mode, the exoskeleton manipulated upper extremity with three-dimensional trajectory predetermined by the therapists according to patient-centered goals. Moreover, with the three-dimensional animation and voice prompts from the exoskeleton, patients were required to pay attention to the left side. During assist-as-need mode, patients practiced games and ADL training programs dedicated to the left side with audiovisual feedback, such as shooting targets, Whack-a-Mole, and cleaning windows. The Armule detected human-robot interaction forces and momentary position via the sensors in the linkage cuffs to estimate the participants' real-time movement intentions and performance for assistance when necessary. Training programs were progressed according to the performance of patients. The difficulty level for USN intervention was changed during robotic training by adjusting where the targets occurred on the computer screen, range of motion, and the robotic assistance. Besides, therapists could regulate the motion sensitivity of the exoskeleton to increase training difficulty for motor function. When the patient was not able to complete the tasks actively, the exoskeleton gave acoustic cues to patients and assistance supplied for the upper extremity supervised by the therapist.

Participants in the CT group received general cognitive and occupational rehabilitation dedicated for USN, consisting of visual scanning therapy, passive range of movement of upper extremity and perceptual retraining integrated with task-specific activities.

Interventions in both groups were delivered at the same frequency, intensity, and duration: 45 min daily, 5 days/week for 4 weeks. The same therapist and therapy assistant deliver all the intervention sessions in the study. Because the participants for both groups required multidisciplinary treatment, conventional rehabilitation programs in the Department of Rehabilitation Medicine continued as usual for all the participants.

### Outcome Measures

Behavioral Inattention Test (BIT). The BIT is a valid and reliable battery (including behavioral section and conventional section) commonly used to assess spatial neglect. In this study, we used the conventional section (BIT-C) with six items: line crossing, letter and star cancellation tests, figure and shape copying, line bisection, and representational drawing. The maximum score is 146 with a cut-off score <128 for spatial neglect. The assessment could be separated into several sessions within 2 days if the participant felt fatigue to the extent of affecting the score (33).

Catherine Bergego Scale (CBS). The CBS is a 10-item scale, which is a validated and reliable measure in daily life performance related to USN, including grooming, dressing, and wheelchair driving. Each item is scored as 0, 1, 2, or 3 to indicate no neglect, mild, moderate, or severe neglect, respectively. Total score is calculated (range, 0–30) with a higher score indicating more severe hemispatial neglect (34, 35).

Fugl-Meyer Assessment for Upper Extremity (FMA-UE). The FMA-UE has good reliability, validity, and responsiveness in stroke patients for motor impairments assessment. The FMA-UE examines arm movement, coordination, and reflexes with results represented in a three-point ordinal scale. The FMA-UE score ranges from 0 to 66, with higher scores indicating fewer motor impairments (36).

Modified Barthel Index (MBI). The MBI is an assessment tool intended to examine the level of independence in the basic ADL including 10 categories: feeding, personal hygiene, toilet use, bladder control, bowel control, bathing, dressing, chair/bed transfer, ambulation, and stair climbing. MBI scores range from 0 to 100, with higher scores indicating the greater degree of functional independence (37).

World Health Organization Disability Assessment Schedule Version 2.0 (WHODAS 2.0). The WHODAS 2.0 is a validated tool to assess six domains of social participation: understanding and communicating, self-care, getting along with people, life activities, and participation in society. These domains cover 36 items regarding the condition of the participants in the last 30 days. Each item is rated on a five-point scale from one (normal) to five (severe disability) (38).

### Statistical Analysis

All statistical analyses were performed using SPSS software (version 21.0, IBM Corporation, Chicago, IL). To assess the normal distribution of quantitative data, Shapiro-Wilk test or Q-Q plot was employed (*P* > 0.05). Baseline data between the groups were compared using independent-samples *t*-tests for continuous variables, and chi-square (χ^2^) tests for categorical variables. Within-group differences were compared using paired *t*-tests for the normally distributed data, and Wilcoxon-signed rank test for the non-parametric equivalent test. To examine the effect of randomization procedure, between-group differences were compared using an independent *t*-test and χ^2^ test for continuous and dichotomous data, respectively. All data were presented as means ± standard deviations in tables unless otherwise stated. For all tests, the level of significance was set at 0.05.

## Results

**Figure 1:**
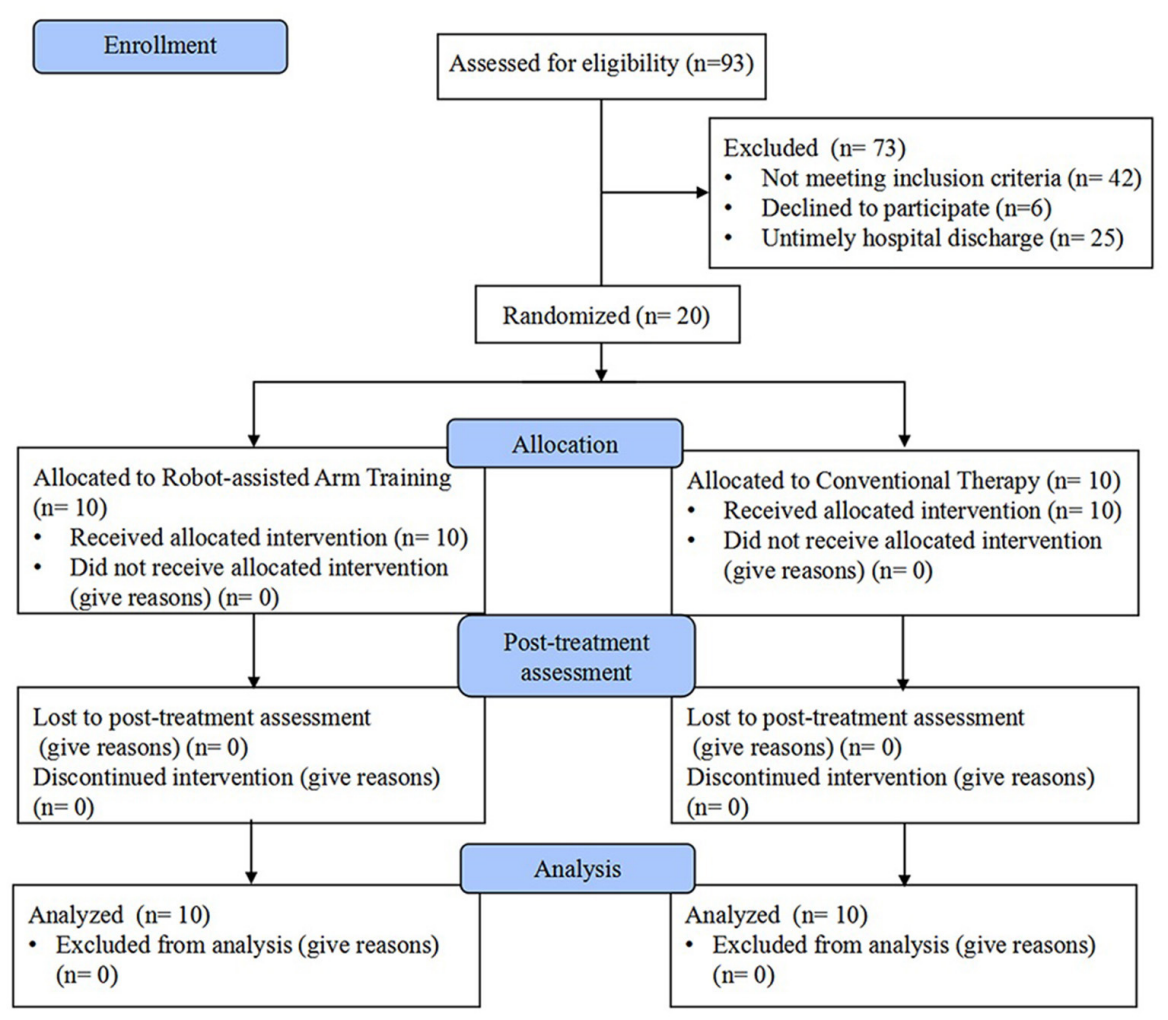
Consolidated Standards of Reporting Trials (CONSORT) flow chart of the study.

From November 2018 to February 2021, stroke patients were screened for eligibility. Twenty patients (mean age 47.40 ± 8.47) with left-sided USN were enrolled in the study and completed the intervention with assessments. A Consolidated Standards of Reporting Trials (CONSORT) flow chart that outlined participant flow through the study is presented in Figure 1. Baseline demographics and clinical characteristics are demonstrated in Table 1, and there were no significant between-group differences at baseline. No participant demonstrated difficulty following the treatment instructions, nor did anyone express complaints or report fatigue, discomfort, or adverse events occurring during the study. Both groups showed significant within-group improvements in all outcome measures (*P* < 0.05) after the 4-week intervention (Table 2).

**Table 1:** Participant characteristics (*N* = 20).

**Characteristics**	**RAT (*N* = 10)**	**CT (*N* = 10)**	***p-Value***
Age (years)	46.20 ± 7.02	48.60 ± 9.95	0.54
Gender (M/F)	8/2	7/3	0.74
Days between onset and enrollment	97.10 ± 84.37	86.40 ± 61.92	0.82
Stroke type (Hemorrhagic/ ischemic)	3/7	1/9	0.48
MoCA (range 0–30)	21.30 ± 1.64	23.20 ± 2.86	0.17
BIT-C (range 0–146)	86.30 ± 30.73	82.50 ± 28.78	0.78
CBS (range 0–30)	12.50 ± 5.62	14.70 ± 5.96	0.41
FMA-UE (range 0–66)	23.10 ± 10.48	20.50 ± 8.02	0.54
MBI (range 0–100)	45.60 ± 13.97	50.40 ± 12.79	0.43
WHODAS-2.0 (range 36–180)	122.10 ± 10.84	124.00 ± 11.43	0.71

*MoCA, Montreal Cognitive Assessment; BIT-C, Behavioral Inattention Test conventional section; CBS, Catherine Bergego Scale; FMA-UE, Fugl-Meyer Assessment for Upper Extremity; MBI, Modified Barthel Index; WHODAS-2.0, World Health Organization Disability Assessment Schedule Version 2.0*.

*Values are means ± standard deviation unless otherwise stated*.

*Independent t-tests for all variables except for gender and stroke type (χ2 test)*.

**Table 2:** Outcome measures for the RAT (*N* = 10) and CT (*N* = 10) groups.

**Outcome measures**	**Group**	**Pre-intervention**	**Post-intervention**	**Within-group differences**	**Mean differences between groups (95% CI)**, ***P*** **Value**	
BIT-C	RAT	86.30 ± 30.73	109.70 ± 28.28	23.40 ± 7.85	7.70 (0.55, 14.85)	0.04^*^
	CT	82.50 ± 28.78	98.20 ± 28.39	15.70 ± 7.36		
CBS	RAT	12.50 ± 5.62	7.10 ± 4.33	−5.40 ± 1.65	−1.30 (−2.89, 0.76)	0.10
	CT	14.70 ± 5.96	10.60 ± 4.95	−4.10 ± 1.73		
FMA-UE	RAT	23.10 ± 10.48	37.70 ± 11.11	13.60 ± 4.70	5.10 (1.52, 8.68)	0.01^*^
	CT	20.50 ± 8.02	30.00 ± 7.90	9.50 ± 2.64		
MBI	RAT	45.60 ± 13.97	74.50 ± 14.73	28.90 ± 14.26	7.90 (−3.27, 19.07)	0.16
	CT	50.40 ± 12.79	71.40 ± 12.65	21.00 ± 8.89		
WHODAS-2.0	RAT	122.10 ± 10.84	98.60 ± 8.70	−23.50 ± 7.58	−7.30 (−12.50, −2.10)	0.01^*^
	CT	124.00 ± 11.43	107.80 ± 11.70	−16.20 ± 6.99		

*BIT-C, Behavioral Inattention Test conventional section; CBS, Catherine Bergego Scale; FMA-UE, Fugl-Meyer Assessment for Upper Extremity; MBI, Modified Barthel Index; WHODAS-2.0, World Health Organization Disability Assessment Schedule Version 2.0; RAT, robot-assisted arm training; CT, conventional therapy*.

*Values are presented as means ± standard deviation unless otherwise stated*.

*Paired t-tests are used for within-group differences and independent t-test for between-group differences*.

* *P < 0.05*.

### Effect of Robot-Assisted Arm Training for Neglect Symptoms

As shown in Table 2, both groups had significant improvements in neglect symptoms assessed by BIT-C; and daily life performance related to USN assessed by CBS. Participants assigned to RAT group had significantly improvements in BIT-C (difference, 7.70; 95% CI, 0.55–14.85, *P* = 0.04) than the CT group. However, the change scores of CBS demonstrated no significance between the groups (difference, −1.30; 95% CI, −2.89 to 0.76, *P* = 0.10).

### Effect of Robot-Assisted Arm Training for Arm Motor Impairment

After the 4-week intervention, the mean Fugl-Meyer scores measuring upper extremity motor impairment for RAT group were better than CT group (difference, 5.10; 95% CI, 1.52–8.68, *P* = 0.01) (Table 2).

### Effect of Robot-Assisted Arm Training for ADL and Social Participation

RAT and conventional training were associated with improvement in ADL with regard to MBI, but no significant difference was found between the groups (difference, 7.90; 95% CI, −3.27 to 19.07, *P* = 0.16). In contrast, participants receiving RAT therapy had significant improvement in social participation on change scores of the WHODAS 2.0 (difference, −7.30; 95% CI, −12.50 to −2.10, *P* = 0.01) than CT group (Table 2).

## Discussion

In previous studies, robot-assisted training providing task-specific training and sensorimotor stimulation has presented efficacy in upper extremity motor recovery, whereas it was unclear in stroke patients with USN. Moreover, robotics devices in these studies were either wrist-hand or planar robot, thus supporting motions with a limited degree of freedom in a planar space. To our best knowledge, the current study is the first pilot study to investigate the effects of exoskeleton-driven RAT, according to the ICF framework, for stroke individuals with USN. We found that RAT using exoskeleton was feasible and safe for stroke patients with left-side USN. The results demonstrated that RAT might have efficacy in USN symptoms, arm motor impairment, and social participation except daily life performance compared with the control group.

Our results regarding neglect symptoms measured by BIT and motor impairment measured by FMA-UE conventional section were in line with that of Varalta et al. (27). They reported that left hand passive movements using a rehabilitation glove for 2 weeks had improvement in neglect assessed by Line Crossing test, Sentence Reading test, and Saccadic Training, and as well as manual dexterity assessed by the Purdue Pegboard test. This indicated that sensory stimulation and human–robot interaction might exert psychological and physiological effects on the neglect symptoms. Although they speculated limb activation therapy as the effective component, there was no control group and merely passive movements distal to the hand in their training programs (27).

Whereas, the preliminary results suggested that improvements of spatial neglect in experimental setting (BIT-C) might not generalize to daily life performance related to USNspatial neglect assessed by CBS, and ADL performance assessed by MBI. These results were contrary to that of Choi et al. who found that RAT via planar robot had no additional benefit for neglect symptoms and ADL in stroke patients compared with conventional neglect treatment (28). USN is a neuropsychological disorder to explore, orient, or respond to the contralesional side of somatic and extrasomatic space commonly involving the right brain hemisphere. A possible explanation for these results might be that our robotic protocol focused on personal and peripersonal space while possibly overlooking the extrapersonal area in USN patients of extrapersonal subtype (39). Besides, the provided training movements were limited in degree of freedom and low-dimension space without tasks related to ADL in the virtual environment. Additionally, ceiling effects of the outcome measures especially in MBI, might have masked the differences between the groups (40).

We used the WHODAS 2.0 to measure patients' performances in understanding and communicating, self-care, getting along with people, life activities, and participation in society. Although both groups improved in all domains of social participation, patients treated with RAT showed better between-group improvements than CT group. Interestingly, Karner and colleagues demonstrated in a RCT that the PARO interactive stimulation robot could improve visuospatial hemineglect measured by the cancellation tests and Cats Test, and social participation measured by the SINGER test (41).

Previous RAT studies were mainly focused on upper extremity motor recovery after stroke (23). Our study extended its application and showed that RAT via exoskeleton might be feasible and safe for stroke patients with left-side USN. Moreover, such stroke population may be beneficial in not only motor impairment measured by FMA-UE but also neglect symptoms from the robotic arm therapy targeted for remediating USN compared with CT. According to previous literature, the reason could be that robotic device consisted of top-down and bottom-up techniques concurrently, although which warranted further investigation. During RAT intervention, therapist encouraged the participants to pay attention to and explore the space contralateral to the brain lesion, which might serve as a visual scanning training for visual searching and object catching (7, 42). Besides, passive movements to multiple joints with the exoskeleton may play a role as limb activation therapy in effect of arousing contralateral attention (17). Finally, under a virtual, feedback-based environment when interacting with the games, patients voluntarily controlled for head, eyes, and limbs movements or postural shifts to explore, orient, or respond to the affected side (43, 44).

## Limitations

There are several limitations warrant caution when interpreting the results of our study. First, the pilot study without fully powered sample size and follow-up assessment limits the validity of our results and the investigation of long-term effects. Randomized clinical trials with larger sample sizes and longer follow-up duration are warranted to confirm these preliminary results. Second, clinical characteristics of the participants, such as neglect subtypes, stroke types, onset time, lesion size, and stroke severity, might make a difference to the results; thus, a larger-scale study with subgroup analysis may help identify the efficacy of RAT (45). Third, the exoskeleton protocol included several intervention ingredients, while evidence needs to determine the effective components of the training programs with underlying neurological mechanisms. Finally, in the present study, patients underwent only behavioral but not neuroimaging assessments. Future studies should investigate possible changes in brain lesion and neurological network after robotic therapy.

## Conclusions

Our findings provide preliminary support for introducing RAT to remediate USN after stroke. RAT that focuses on neglect of contralateral space and affected upper extremity may be effective in motor function recovery and social participation, while not generalizing into improvements in ADL. Future researches with larger sample sizes need to investigate the effects of RAT for various patients and the underlying neurological mechanisms.

## Data Availability Statement

The raw data supporting the conclusions of this article will be made available by the authors, without undue reservation.

## Ethics Statement

The studies involving human participants were reviewed and approved by The Clinical Trials Ethics Committee of Huazhong University of Science and Technology. The patients/participants provided their written informed consent to participate in this study.

## Author Contributions

ZJC and XLH conceived the study design. ZJC, MHG, and CH performed the clinical trials. ZJC and CH analyzed the results. ZJC drafted the manuscript. All authors contributed to manuscript revision, read, and approved the submission of the manuscript.

## Conflict of Interest

The authors declare that the research was conducted in the absence of any commercial or financial relationships that could be construed as a potential conflict of interest.
